# Myocarditis Secondary to Mesalamine-Induced Cardiotoxicity in a Patient with Ulcerative Colitis

**DOI:** 10.1155/2018/9813893

**Published:** 2018-03-15

**Authors:** Kelechukwu U. Okoro, Matthew D. Roby, Adegbenga A. Bankole

**Affiliations:** ^1^Department of Internal Medicine, Virginia Tech Carilion School of Medicine and Research Institute, Roanoke, VA, USA; ^2^Department of Internal Medicine, Section of Cardiology, Virginia Tech Carilion School of Medicine and Research Institute, Raonoke, VA, USA; ^3^Department of Internal Medicine, Section of Rheumatology, Virginia Tech Carilion School of Medicine and Research Institute, Raonoke, VA, USA

## Abstract

Development of cardiac manifestations in patients diagnosed with inflammatory bowel disease undergoing treatment with mesalamine is a rare. When this occurs, it can be difficult to tease out the primary etiology, as both IBD and mesalamine can cause cardiac manifestations independently of each other. The exact mechanism of mesalamine-induced cardiotoxicity is yet to be determined although several mechanisms have been described. We present the case of a gentleman with nonexertional chest pain in the setting of ulcerative colitis exacerbation believed to have occurred secondary to mesalamine.

## 1. Introduction

Ulcerative colitis (UC) is one of the two subtypes of inflammatory bowel disease (IBD). It is a chronic inflammatory disease of the colon whose etiology is unknown but it is thought to occur via an autoimmune mechanism. It usually presents with abdominal discomfort, hematochezia, and diarrhea. UC is also known to cause extraintestinal manifestations such as uveitis and erythema nodosum. At times, UC may affect the heart causing pericarditis or myocarditis. Patients with UC are usually prescribed mesalamine which can also cause cardiac complications. Cardiotoxicity secondary to mesalamine usually improves with withdrawal of the medication while UC cardiotoxicity improves with adequate disease control. When these two entities occur together, it is challenging to tease out the primary etiology.

## 2. Case Presentation

A 23-year-old Caucasian gentleman whose past medical history was significant for ulcerative colitis (UC), migraine without aura, allergic rhinitis, grade 3 obesity, obstructive sleep apnea, hypothyroidism, prediabetes, seizure disorder, and gastroesophageal reflux disease presented to the emergency department complaining of symptoms of UC flare including nausea, vomiting, abdominal pain, bright red blood per rectum, and about ten bowel movements a day for approximately seven days. Review of systems was notable for dizziness, palpitations, and chest pain which had been ongoing for some time. The patient reported that he experiences intermittent, substernal, 8 out of 10, crushing left-sided chest pain radiating to his left mandible and left upper extremity. The patient stated that the pain was not exacerbated by activity but noted that whenever he started a steroid taper for his UC, the chest pain resolved only to recur when the taper was stopped. Current medications at time of visit to the ED included mesalamine and Levothyroxine.

**Figure 1:**
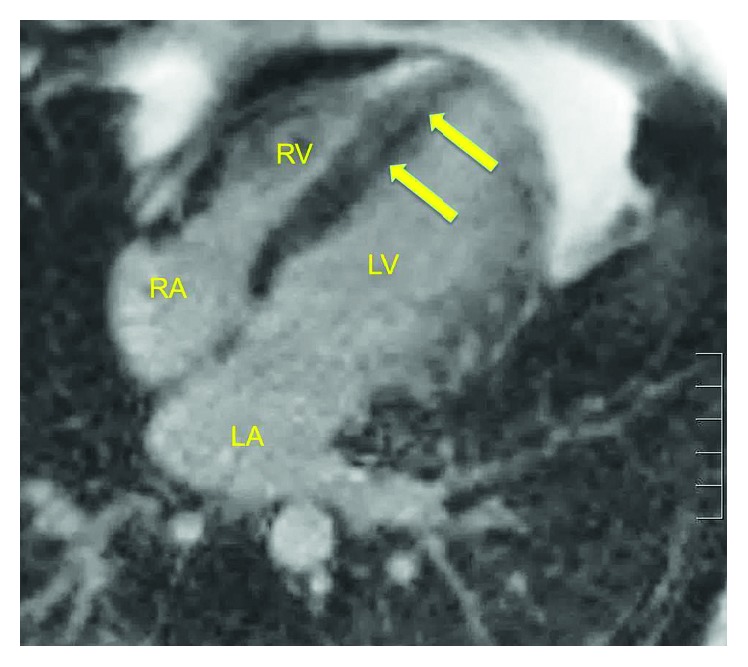
Magnitude reconstructed inversion recovery image of MRI T1-weighted imaging depicting linear delayed hyper-enhancement involving the midmyocardium of the distal septum. This translates to gadolinium lighting up in the midmyocardium due to loss of cellular integrity.

**Figure 2:**
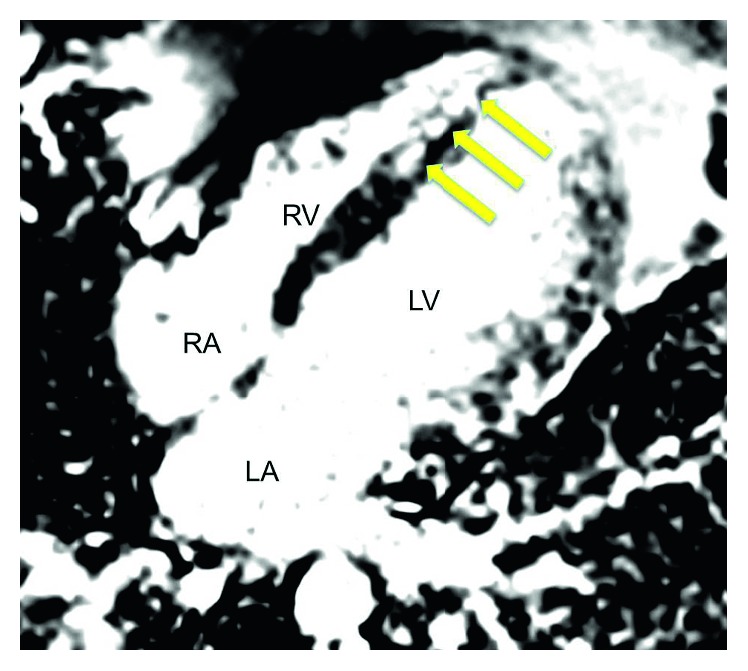
Phase sensitive inversion recovery image depicting delayed hyper-enhancement involving the midmyocardium with endocardial sparing.

Physical examination was normal except for tachycardia and abdominal pain to palpation without rebound, guarding, or rigidity. Labs were grossly normal except hemoglobin 12.3 g/dl, hematocrit 37.7, sedimentation rate 96 mm/hr, C-reactive protein 20.16 mg/dl, and troponin 14.55 ng/dl. EKG revealed sinus tachycardia without ischemic changes. CT angiogram of the chest was unrevealing. Transthoracic echocardiography (TTE) demonstrated an akinetic apex, but overall preserved ejection fraction. A cardiac magnetic resonance imaging (cMRI) was ordered due to the patient's atypical presentation, young age, and lack of traditional risk factors for coronary artery disease. MRI revealed linear delayed hyper-enhancement involving the midmyocardium of the distal septum suspicious for myocarditis (Figures 1 and 2). The patient's mesalamine was discontinued, and he was started on Methylprednisolone. His condition improved, and he was discharged from the hospital with Adalimumab as his primary UC therapy.

## 3. Discussion

Inflammatory bowel disease (IBD) as the name implies is an inflammatory condition that affects the gastrointestinal tract. It is mainly divided into two subtypes, ulcerative colitis and Crohn's disease. IBD may also affect a variety of extraintestinal organs such as integumentary, musculoskeletal, and pulmonary organs. Extraintestinal manifestations are common and occur in approximately 25–30% of patients although cardiac involvement is rare [1–3]. Manifestations of cardiac involvement include pericarditis, myocarditis, and myopericarditis.

Mesalamine is an aminosalicylate anti-inflammatory drug commonly prescribed in patients with IBD. The exact mechanism of the drug is unknown but it is thought to inhibit prostaglandin formation by inhibition of cyclooxygenase (COX) enzyme thereby decreasing signaling via PPAR-γ pathway leading to decreased activity of nuclear factor ĸB. Inhibiting this cascade leads to decreased colonic inflammation [1]. Common adverse effects of mesalamine include nausea, abdominal discomfort, headache, and fatigue. An additional side effect of mesalamine that has not been extensively described in literature is cardiotoxicity. The drug can cause pericarditis, myocarditis, and coronary vasculitis [3]. The specific mechanism of mesalamine-induced cardiotoxicity is yet to be described, but several hypotheses have been proposed. The first is accelerated metabolism of arachidonic acid to lipoxygenases secondary to inhibition of COX enzyme. This leads to the overproduction of eosinophil-stimulating cytokines thereby initiating a hypersensitivity reaction [4]. Another proposed mechanism involves humoral-mediated hypersensitivity, in which antibodies formed against mesalamine cross react with cardiac tissues causing inflammation [1, 3]. Other hypothesized mechanisms include a direct toxic effect of mesalamine on the myopericardium, a cell-mediated hypersensitive reaction, and an allergic reaction mediated by immunoglobulin E [4, 5]. Irrespective of the mechanism, it is very important to be aware of its cardiotoxic effects as symptomatology may range from benign chest discomfort to florid heart failure and cardiogenic shock. Symptoms usually begin within 2–4 weeks after initiation of the drug although presentation may be delayed due to concomitant use of steroids [3].

There are no physical findings, symptoms, or laboratory tests that are pathognomonic for mesalamine-induced cardiotoxicity [3]. The diagnosis is supported by relatively early onset of symptoms once the drug is begun, resolution of symptoms within one week after withdrawal, and by worsening of symptomatology once the drug is reintroduced in the acute phase [3]. This is in contrast to IBD cardiotoxicity which usually occurs years after initial diagnosis although it may occur on initial presentation [5, 6]. UC cardiotoxicity is usually a diagnosis of exclusion after connective tissue disorders, malignancy, infection, and metabolic causes have been ruled out [1]. Our patient presented with cardiac symptoms 6 months after initial diagnosis of UC. At time of initial diagnosis, he had no cardiac complaints. He was started on Mesalamine 800 mg PO TID along with 60 mg of prednisone which was to be tapered by 5 mg each week for 12 weeks. His 5 mg taper was decreased to a 10 mg taper, and he completed the regimen. His mesalamine formulation was switched to 2.4 mg daily of a long-acting preparation for easier dosing. 4 months later, he developed acute exacerbation of UC, requiring him to restart steroids and prompting his mesalamine dose to be increased to 4.8 mg daily. This was not sufficient with the patient having to report to the ED the following day.

The patient could not remember the exact onset of his chest pain but he did note that the pain improved with initiation of steroids and worsened or became more apparent whenever he tried to taper the steroids. We suspect the delayed presentation of myocarditis to be secondary to the coadministration of mesalamine and steroids. Mesalamine was discontinued, and the patient was started on methylprednisolone with eventual resolution of both chest discomfort and UC symptoms. He was started on adalimumab after discharge and has had no further cardiac complaints. He has continued to experience UC flares with associated spondyloarthropathy.

## 4. Conclusion

It is imperative to consider myopericarditis in patients with IBD being treated with mesalamine as both may cause severe cardiotoxicity with development of heart failure symptoms. In the case of mesalamine-induced cardiotoxicity, no specific therapy is needed as prompt withdrawal of the drug leads to rapid amelioration of symptoms although administration of steroids is not a frowned upon alternative. Steroid administration is the first choice of management in patients with IBD cardiotoxicity.
